# Is Green Spread? The Spillover Effect of Community Green Interaction on Related Green Purchase Behavior

**DOI:** 10.3390/ijerph19116571

**Published:** 2022-05-27

**Authors:** Jianming Wang, Xincheng Yang, Yini Xi, Zhengxia He

**Affiliations:** 1 School of Business Administration, Zhejiang University of Finance & Economics, Hangzhou 310018, China; sjwjm@zufe.edu.cn (J.W.); yangxincheng1226@163.com (X.Y.); zcxyn@zufe.edu.cn (Y.X.); 2 School of Economics, Hangzhou Normal University, Hangzhou 311121, China

**Keywords:** community green interaction, environmental emotion, related green purchase behavior, spillover effect, social diffusion mechanism

## Abstract

In the era of digital economy and mobile internet, many platforms or brands have built various online or offline green communities to guide customers or fans to engage in green interactions. Obviously, community green interaction can enhance brand emotional value and enhance customer stickiness, but whether community green interaction can further have a spillover effect on related or other green purchase behaviors has become an important topic for the theoretical and practical departments. This paper selects the “Little Bear Fuel Consumption Community” as the research object. Based on the theoretical framework of “Green Interaction—Environmental Emotion—Related Green Purchasing Behavior”, this paper examines the spillover effect and impact mechanism of community green interaction on consumers’ related green purchasing behavior. This paper uses a structural equation model and bootstrapping method to test the causal relationship between variables. This study lasted for 6 months, and a total of 348 valid questionnaires were collected in this study. We used SPSS 25 and AMOS 24 for data analysis. The results showed that the two dimensions of community green interaction (community green information interaction and community green interpersonal interaction) have a positive spillover effect on consumers’ related green purchase behavior; community green interaction can positively spill over to consumers’ related green purchase behavior through the psychological path of environmental emotion; community green information interaction and community green interpersonal interaction have positive effects on consumers’ positive and negative environmental emotions; positive and negative environmental emotions positively affect consumers’ related green purchase behavior; and in the two paths of community green information interaction—related green purchase behavior and community green interpersonal interaction—related green purchase behavior, both positive environmental emotion and negative environmental emotion play a role of partial mediation; product involvement has a negative moderating effect on the path of “community green interaction—environmental emotion”. This paper opens the “black box” of the diffusion mechanism of community green interaction and provides a new explanatory framework for the spillover effect of community green interaction on related green purchase behavior.

## 1. Introduction

In the era of digital economy and mobile Internet, many platforms or brands have built diverse communities. In the traditional brand community, consumers or users often interact on products, services, and industries. Studies have confirmed that community interaction can improve consumers’ loyalty, satisfaction [1,2,3], product purchase intention [4], value co-creation [5], and product innovation behavior [6]. With the development of the industry and the increasing attention of enterprises to green environmental protection, more and more enterprises begin to build a brand green community. For example, Patagonia has launched Patagonia Action Works, a digital platform de-signed to connect environmental volunteers with environmental activists. The platform is similar to an “online social networking site”. After entering the platform, users can enter the name of their area, and then search for corresponding environmental protection organizations based on different information, such as land, water, climate, community, biodiversity, and species protection. In the brand green community, consumers often interact on the topics of resource conservation, green products, green industry exhibitions, and so on. With the development of mobile Internet technology, there are more and more cases in which consumers pay attention to and share green consumption information on brand green community or other social media platforms, so as to drive more people to participate in the follow-up related green purchase behavior. However, at present, such community green interaction and its influence have not been paid enough attention in the academic circles. Obviously, community green interaction can enhance brand emotional value and enhance customer stickiness, but will this kind of community green interaction make consumers’ subsequent or other related purchase behavior “greener”? This is a topic worthy of attention and discussion.

The relationship between community green interaction and associated green purchase behavior belongs to the research category of spillover effect. Research on behavior spillover effect focuses on the causal relationship between consumers’ previous and subsequent behaviors, that is, the impact of initial behavior on subsequent behavior. For example, residents tend to buy more green products in subsequent consumption after garbage classification. Studies have proved that water saving, electricity-saving behavior, residents’ recycling, reducing the use of hotel towels, reducing fuel consumption, and recycling packaging bags have affected consumers’ subsequent environmental behavior [7,8,9,10], and even have affected consumers’ support for some environmental policies [11,12,13,14]. However, most research is limited to the study of some behaviors that consumers can complete independently, such as water saving, waste classification, etc.; behaviors that need to interact with others, such as community interaction, have not received extensive attention at present. In addition, most studies related to spillover effects focus on two directly related behaviors of consumers (such as waste recycling behavior and energy-saving behavior) and lack of exploration on the spillover effects and their mechanism between green interaction and related green behaviors. Based on the theoretical perspectives of spillover effect and social diffusion, this study aims to explore the following four issues: (1) the direction of the spillover effect of community green interaction on related green purchase behavior. That is, after participating in community green interaction, do consumers prefer to make subsequent green purchases or are they more reluctant to make subsequent green purchases? (2) The strength of the spillover effect of community green interaction on related green purchase behavior. That is, the impact of consumers’ participation in community green interaction on consumers’ related green purchase behavior in their daily life. (3) The diffusion mechanism of the spillover effect of community green interaction on related green purchase behavior. That is, what is the mechanism of community green interaction affecting related green purchase behavior? (4) The boundary condition of the spillover effect of community green interaction on related green purchase behavior. That is, under what conditions does the spillover effect of community green interaction on related green purchase behavior hold?

The structure of the rest of this paper is as following: Section 2 is theoretical basis and research model; Section 3 is about research methods and sample analysis, which contains participants, instrument, data collection and description, reliability and validity test, and common method deviation test; Section 4 is data analysis and empirical results (including model fitness and path coefficient test, test of mediating effect of environmental emotion, and test of moderating effect of product involvement); Section 5 is discussion; and the final section, Section 6, is conclusions, which includes theoretical contributions, management implications, research limitations, and future prospects.

## 2. Theoretical Basis and Research Model

### 2.1. Theoretical Basis

#### 2.1.1. Spillover Effect Theory

Scholars have defined the spillover effect in environmental problems as the impact of participating in a behavior on the probability of subsequent behavior. Existing studies mainly divide spillover effects into behavior spillover, time spillover, and environment spillover. Behavior spillover refers to behavior A leading to behavior B, which is the most common type of spillover in previous studies [15,16]. Time spillover focuses on how to formulate a behavior conducive to the environment to affect the frequency of the same behavior in the future, that is, how a behavior changes with the change of time or environment [17]. Environmental spillovers focus on how specific behaviors spread across the environment [18,19]. From the perspective of results, spillover effects can be divided into positive spillover and negative spillover. Positive spillover means that participating in the first behavior will increase the possibility of participating in the second behavior. For example, Xu et al. confirmed that recycling has a positive spillover effect on consumers’ green consumption [20]. Negative spillover effect means that participating in the first behavior will reduce the possibility of participating in the second behavior [16]. For example, the laboratory experimental results of Chatelain et al. (2018) support negative spillovers between residents’ private environmental protection behaviors [21]. Ma et al. found that after consumers are forced to make pro environmental behavior, they will later make behavior that destroys the environment [22].

#### 2.1.2. The Theory of Cognitive Dissonance

The theory of cognitive dissonance was put forward by Fistinger in 1957. This theory holds that the individual’s cognitive structure is composed of many cognitive elements, such as thought, concept, attitude, own behavior, and so on. If there is disharmony or conflict between cognitive elements, cognitive imbalance will occur, which will make individuals feel uncomfortable. In order to eliminate such negative psychological state, individuals have three means: choose to change one of the elements to maintain cognitive consistency; add new cognition; or emphasize the importance of one of them [23]. The theory of cognitive dissonance can be used to explain the generation of positive spillover effect. From the perspective of cognitive dissonance, the spillover effect of positive green behavior occurs because people want to avoid the unpleasant feeling of inconsistent performance between different pro environmental behaviors.

#### 2.1.3. Self-Perception Theory

Self-perception can mainly be used to explain the impact of behavior on self-cognition. When people form evaluation cognition (such as attitude, norms, and values), they will take their own behavior as a clue [24], that is, people will understand their attitude, emotion and psychological state according to their own behavior and the situation in which the behavior occurs. A key point of self-perception theory is that behavior comes before attitude, that is, there is behavior first, then emotion, and then further cognition [25]. This theory can be used to explain the spillover effect of pro-environmental behavior. After making behaviors related to environmental protection, consumers will further judge their attitude towards environmental protection, so they are more likely to engage in pro environmental behaviors consistent with their self-perception in subsequent behaviors.

#### 2.1.4. Social Diffusion Theory

Social diffusion theory originates from innovation diffusion theory, which originally refers to the process in which new technologies and products diffuse from innovation providers to social systems over time and are gradually applied or accepted by potential adopters [26]. With the passage of time, the innovation diffusion theory is not only limited to the fields of new technologies and new products, but also gradually applied to the fields of policy innovation diffusion and consumption behavior innovation diffusion [27]. In the era of digital economy and mobile Internet, with the help of mobile Internet technology, innovative consumption concepts and behavior patterns quickly penetrate through brand communities or other social media platforms, reflecting the “diffusion effect” and “herding effect” in the field of consumption, which shows that consumers influence their purchase decisions by observing others’ consumption behavior and learning online interactive information [28].

#### 2.1.5. Research Framework

At present, there are few studies on community interaction in the field of green consumption. However, it is found that “interaction-psychological change-response” is a research framework suitable for community interaction. Based on the research framework of “customer interaction—customer emotion—post purchase satisfaction”, Jing et al. explored the influence mechanism of customer interaction on consumers’ post purchase satisfaction [29]. Based on the path of “customer interaction-self-determination-community satisfaction”, Wang et al. analyzed the role of self-determination in customer interaction and community satisfaction [30]. Therefore, this study adopts the framework of “interaction- psychological change- response” for the construction of theoretical model.

### 2.2. Literature Review and Research Hypothesis

#### 2.2.1. Community Green Interaction

Community interaction mainly refers to the communication among community individuals [31]. Interaction is essentially the exchange of information between communicating individuals [32], and the community is the platform for consumers to communicate and interact. Community interaction enables consumers to establish contact with other members of the community, and makes consumers’ understanding of products more comprehensive and three-dimensional through continuous communication and exchange [33]. Nowadays, with the upgrading of green consumption, more and more communities take green environmental protection as their interactive content. To sum up, this study proposes that community green interaction mainly refers to the interaction of community members around resources saving and environmental protection. Previous studies confirmed that community interaction can affect consumers’ purchase intention, purchase decision, purchase behavior, and repeated purchase intention [4,33,34,35]. Compared with the traditional community interaction, community green interaction pays more attention to environmental protection, showing the characteristics of pro-environment and pro-society, so it may be closely related to consumers’ related green purchase behavior. Combined with previous studies on the impact of community interaction on consumers, this study believes that community green interaction may spill over to consumers’ related green purchase behavior.

#### 2.2.2. Dimension of Community Green Interaction

Scholars have proposed a variety of ways to divide the dimensions of community interaction from different perspectives. Based on the starting point of community green interaction, this study believes that members participate in green interaction mainly for two purposes: one is to obtain professional information related to green products, the use of green products, and the recent development of green industry; the second is to establish emotional contact with other members of the community through interaction. Therefore, referring to the division method of Jing et al. (2013), this study divides the community green interaction into two dimensions: green information interaction and green interpersonal interaction [29]. Green information interaction is an interaction based on the topic of enterprise green products and industries; green interpersonal interaction is an interaction based on the topics of resource conservation, environmental protection, and daily life, and it mainly focuses on interpersonal communication and exchange among members, rather than professional green information sharing and discussion.

#### 2.2.3. Spillover Effect of Community Green Interaction on Consumers’ Related Green Purchase Behavior

First of all, after participating in the community green information interaction, on the one hand, consumers can obtain professional information about green products, industries, energy conservation, and environmental protection; on the other hand, they can share their own green information with other members of the community. In the process of constantly exchanging information, consumers’ green cognition will be continuously improved and strengthened. After participating in the green interpersonal interaction of the community, consumers have established a close relationship with other members, and their attitudes and concepts on environmental protection will be more vulnerable to the influence of other members. To sum up, participating in community green interaction (information interaction and interpersonal interaction) will improve consumers’ cognition and attitude. Further, according to the theory of cognitive dissonance, perceived inconsistencies between cognitive or behavioral elements will lead to uncomfortable feelings [36]. This discomfort, in turn, stimulates dysregulation reduction strategies, such as behavior change, or the balance of the two behaviors [37]. Therefore, in order to maintain cognitive consistency and avoid the unhappiness caused by cognitive imbalance, consumers participating in community green interaction will be more inclined to related green purchase behavior, that is, community green interaction has a positive spillover on consumers’ related green purchase behavior.

Secondly, according to self-perception theory, people will know themselves according to their behavior and the situation in which the behavior occurs. As the theme of community green interaction is to protect the environment and reduce resource waste, it can be regarded as a pro environmental behavior to a certain extent. After consumers communicate and discuss in the green community, they will have environmental self-identity. Environmental self-identity refers to the degree to which individuals regard themselves as environmentalists. Individuals with strong environmental self-identity are more likely to save resources and reduce waste generation [38]. In other words, after participating in the community green interaction, consumers will have the self-cognition of “I am an environmental protection person” and “I am a green consumer”, and think that they have the responsibility to protect the environment and save resources, so as to match their own behavior with their own environmental protection identity, and they are more likely to carry out related green purchase behavior in the future.

Finally, consumers’ participation in community green interaction is often regarded as spontaneous behavior. According to attribution theory, if individuals attribute the initial behavior to internal causes, it is more likely to produce positive spillover. That is, consumers will regard green interactive behavior as something they take the initiative to do, rather than due to the promotion of the external environment. Therefore, compared with negative spillover, community green interaction is more likely to have positive spillover on subsequent related green purchase behavior. To sum up, this study puts forward the following hypotheses:

**H1a.**  *Community green information interaction has a positive spillover effect on consumers’ related green purchase behavior.*

**H1b.**  *Community green interpersonal interaction has a positive spillover effect on consumers’ related green purchase behavior.*

#### 2.2.4. Community Green Interaction and Environmental Emotion

Environmental emotion refers to people’s sensitivity to the significance of saving resources and protecting the environment, the waste of resources and the pollution of the environment, or the emotion expressed by people when participating in environmental protection actions and the subsequent attitude experience [39]. Wang (2015) explored the structural dimension of environmental emotion through qualitative research and found that environmental emotion is divided into positive dimension and negative dimension [40]. Specifically, positive environmental emotion refers to the feelings of pleasure, pride, approval, and love for the improvement of environmental problems or the implementation of better environmental behaviors, while negative environmental emotion refers to the feelings of guilt, worry, anger, and hatred for the deterioration of environmental problems or the implementation of worse environmental behaviors.

This study argues that participating in community green interaction can improve consumers’ environmental emotion. First, community green information interaction can effectively improve consumers’ green cognition. Community green information interaction can enable consumers to have more professional environmental knowledge, such as which products are more beneficial to the environment and which behaviors can lead to better resource saving. Meanwhile, community green information interaction can also make consumers have strong environmental awareness and have high sensitivity to environmental issues. According to the “cognition-emotion-behavior” model in psychology, cognition is the antecedent variable of emotion, and individual cognition of external things and stimuli produces related emotions [39,41,42]. The level of green cognition can be improved through community green information interaction, and then the environmental emotion will be further enhanced. Therefore, consumers will have a stronger sense of pleasure (approval) for their (others’) good environmental behavior, that is, they have a strong positive environmental emotion; conversely, consumers will have a strong sense of guilt (worrying) about their (others’) bad environmental behavior, that is, they have a strong negative environmental emotion. Accordingly, this study puts forward the following hypotheses:

**H2a.**  *Community green information interaction positively affects the positive environmental emotion of community members.*

**H2b.**  *Community green information interaction positively affects the negative environmental emotion of community members.*

Secondly, compared with the cognitive improvement brought by community green information interaction, participating in community green interpersonal interaction will affect consumers’ attitudes and concepts to a greater extent, and then improve environmental emotion. Social identity theory holds that when an individual realizes that he belongs to a specific social group, he will also realize the emotional and value significance brought to him as a group member. The awareness of belonging to a certain group strongly affects our perception, attitude, and behavior, and we endow ourselves with the characteristics in line with the group [43]. Community green interpersonal interaction can enhance the mutual trust and intimacy of community members [30,44], and make community members gain a sense of identity [45] and belonging. After having a sense of identity with the community, consumers’ attitudes and emotions are more likely to be affected by the green values of the community, and then have stronger environmental emotions. Therefore, participating in community green interpersonal interaction makes consumers have higher positive environmental emotion and negative environmental emotion.

**H3a.**  *Community green interpersonal interaction positively affects the positive environmental emotion of community members.*

**H3b.**  *Community green interpersonal interaction positively affects the negative environmental emotion of community members.*

#### 2.2.5. The Mediating Role of Consumers’ Environmental Emotion

Different from emotion, environmental emotion is a lasting and stable emotion, so the same consumer can have positive and negative environmental emotion at the same time, which can change consumers’ purchase behavior to a certain extent [46,47]. Wang (2015) verified that the two dimensions of environmental emotion (positive emotion and negative emotion) have a positive impact on consumers’ low-carbon purchase behavior [40]. Koenig-Lewis et al. (2014) showed that positive environmental emotion and negative environmental emotion play a complete mediating role in the impact of cognitive benefits on purchase behavior [48]. He et al. (2013) found that green emotion plays a mediating role in the path of green cognition affecting consumer behavior [39]. To sum up, this study infers that the two dimensions of environmental emotion (positive emotion and negative emotion) can positively affect consumers’ related green purchase behavior. Combined with the impact of community green interaction on environmental emotion, this study believes that environmental emotion plays a mediating role between community green interaction and related green purchase behavior. Therefore, this study puts forward the following hypotheses:

**H4a.**  *Positive environmental emotion plays a mediating role between community green information interaction and consumers’ related green purchase behavior.*

**H4b.**  *Positive environmental emotion plays a mediating role between community green interpersonal interaction and consumers’ related green purchase behavior.*

**H4c.**  *Negative environmental emotion plays a mediating role between community green information interaction and consumers’ related green purchase behavior.*

**H4d.**  *Negative environmental emotion plays a mediating role between community green interpersonal interaction and consumers’ related green purchase behavior.*

#### 2.2.6. Moderating Effect of Product Involvement

Product involvement mainly explores the subjective psychological state of consumers according to their understanding of products. Product involvement will have an impact on consumers’ information collection and processing behavior. According to the possibility model of fine processing, using different information processing paths (edge path vs. central path) will affect consumers’ decision-making. Consumers with higher product involvement tend to choose the central path to process information. At this time, they will pay more cognitive efforts and pay more attention to the gains and losses brought by green consumption and its impact on society. Consumers with low product involvement tend to choose the edge path to process information, and consumers pay more attention to the feelings related to green consumption, such as pride, appreciation, guilt, contempt, and so on. Therefore, this study argues that community green interaction can better cause environmental emotional changes of consumers with low product involvement. In comparison, since consumers with high product involvement focus on what knowledge information interaction can bring, community information interaction has a low promotion effect on environmental emotion. In addition, community interpersonal interaction cannot bring consumers with high product involvement the information they want to obtain, so its impact on environmental emotion is also small. Therefore, this study puts forward the following hypotheses:

**H5a.**  *Product involvement plays a negative moderating role between community green information interaction and positive environmental emotion.*

**H5b.**  *Product involvement plays a negative moderating role between community green interpersonal interaction and positive environmental emotion.*

**H5c.**  *Product involvement plays a negative moderating role between community green information interaction and negative environmental emotion.*

**H5d.**  *Product involvement plays a negative moderating role between community green interpersonal interaction and negative environmental emotion.*

### 2.3. The Research Model

**Figure 1:**
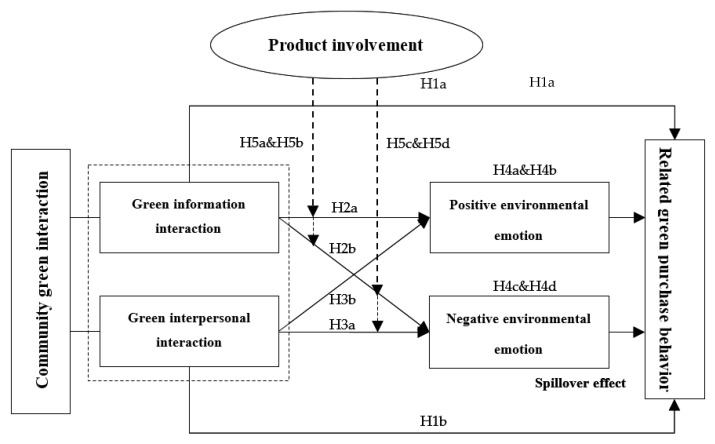
Hypothetical model of spillover effect of community green interaction.

To sum up, based on the theoretical perspectives of spillover effect, self-perception, behavioral attribution, and social diffusion, this study takes community green interaction as the starting point and pays attention to whether it will have behavioral spillover effect on consumers’ related green purchase behavior. According to the characteristics of community green interaction, community green interaction is divided into green information interaction and green interpersonal interaction as antecedent variables. Consumers’ positive environmental emotion and negative environmental emotion are taken as mediating variables to explain consumers’ psychological changes. Consumers’ related green purchase behavior is regarded as the outcome variable reflecting consumers’ response. This study constructs a theoretical framework of community green interaction (information interaction and interpersonal interaction)—environmental emotion (positive environmental emotion and negative environmental emotion)—related green purchase behavior. The hypothetical model examined in this study is shown in Figure 1.

## 3. Research Methods and Sample Analysis

### 3.1. Sample Selection

Considering the typicality of community green interaction, this study selects all consumers who have joined “little bear fuel consumption community” as the research sample. Little bear fuel consumption APP is a kind of vehicle fuel consumption calculation tool, which can accurately assist users in calculating single fuel consumption and average fuel consumption, and make statistics on single month fuel cost and average fuel cost, so as to help users save fuel and money and reduce emission. Users can join the little bear fuel consumption community and participate in interaction, share fuel saving information and daily environmental protection behaviors in the community. The samples we selected are all consumers who have joined the bear fuel consumption community for a period of time and have had community interaction experience.

### 3.2. Measurement

This study used a questionnaire to measure the construct, and the questionnaire consisted of three parts. In the first part, we inform the respondents that this survey is completely anonymous, the information will not be leaked, and the results are only used for academic research. At the same time, we emphasize that this questionnaire is only for members who participate in the green interaction of the bear fuel consumption community. The second part is the scale that measures the construct. The scales used in this study were all seven-point Likert scales, and the measurement items were adapted from the scales of previous studies and adjusted according to Chinese semantics. The measurement of community green information interaction and community green interpersonal interaction refers to the relevant scale of Nambisan and Baron [49], and five measurement items are selected for measuring these constructs respectively; the measurement of positive environmental emotion and negative environmental emotion refers to the research of Steenkamp [50], and six items are selected, respectively. The related green purchase behavior in this study is not limited to the automobile industry, but extended to other consumption fields based on the spillover effect theory. And combined with the scale of existing literature, a total of six items are designed. Product involvement was modified by referring to the scale of Laurent and Kapferer [51]. After the expert group discussion and the data from pre investigation, a formal research scale was finally formed. The items were all on Likert seven scale, “1” means totally disagree, “7” means totally agree. The specific measurement items are shown in Table 1. The third part is the collection of basic information on participants, including demographic variables such as gender, age, education, etc.

### 3.3. Data Collection and Description

Before the formal experiment, an online pre investigation was conducted on the members of the little bear fuel consumption community. A total of 100 questionnaires were distributed, 13 invalid questionnaires were deleted, and 87 valid questionnaires were obtained. The pre investigation results showed that the scale used to measure each construct has good reliability and validity. In the formal survey, the questionnaire was distributed to the members of the community. We have distributed online questionnaires on the Questionnaire Star online platform (https://www.wjx.cn/, accessed on 15 April 2022), which can restrict IP access and prevent respondents from repeating the questionnaire. The questionnaire was shared to the community through WeChat, QQ and other software or filled in by individuals. Participants can open the online questionnaire by clicking a link via mobile devices and terminate or quit at any time when filling in the questionnaire. The samples we selected are all members of the bear fuel consumption community and have had experience in participating in community interactions.

The different communities have been established in the little bear fuel consumption community according to different models, and car owners can interact with others for their models in the community. The small number of people in the community of some unpopular models leads to a poor interactive atmosphere. Therefore, after market research, we selected the 20 most popular models on the market and distributed questionnaires in the corresponding groups. From June to July 2020, we observed members in 20 communities and recorded members who had a record of interacting within the group (a total of 1328 people participated in the interaction). We excluded samples with fewer than two interactions or low participation (e.g., members with very few words spoken), resulting in a final sample of 954. We contacted the above-mentioned members and distributed questionnaires, which they filled out voluntarily. We ensured that a minimum of 20 samples (About 40–50% of the total community) were collected for each community. Therefore, our sample can better represent consumers who participate in the interaction of the little bear fuel consumption community. A total of 400 questionnaires were recovered, we eliminated the questionnaires with too short or too long answering time, and eliminated the questionnaires with too many choices of the same option. In the end, 348 valid questionnaires were obtained, with an effective rate of 87%. Our sample size accounts for 36.4% of the active users of the bear fuel consumption active community. From a data analysis point of view, the sample size required to use the structural equation model is 10–15 times the measurement indicators of the questionnaire. In this study, our measurement index is 31, and the sample size of 348 is sufficient. The descriptive statistics of samples are shown in Table 2. The proportion of men in the total sample was 63.8%, which was attributed to the fact that more men used cars than women. The number of community members aged 25–34 is the largest, mainly because these people are relatively young and willing to interact with others on the community platform. Our sampling mainly covered the central and eastern regions of China (this is the region with relatively developed economy and green development in China), and consumers in these regions are also most likely to engage in community green interactions. Therefore, our samples can better represent consumers who engage in community green interaction in China.

### 3.4. Reliability and Validity Test

#### 3.4.1. Reliability Test

As shown in Table 3, the coefficients Cronbach’s α of each scale ranged from 0.866 to 0.896, all higher than 0.7, indicating that the scale has good reliability.

#### 3.4.2. Content Validity

This study mainly draws on the more mature scales in the literature, which are modified according to the results of expert and group discussion, combined with the research object, background, and purpose, so the scale of this study has good content validity.

#### 3.4.3. Convergent Validity

Using AMOS 24.0 for confirmatory factor analysis, the following test results are obtained: absolute fitness index $\frac{\chi^{2}}{df}$ = 1.178 < 3, RMSEA = 0.023, GFI = 0.915, AGFI = 0.900. value-added fitness index NFI = 0.921, TLI = 0.986, CFI = 0.987, indicating that the fitness of the model is good. As shown in Table 2, the standardization factor loadings of each measurement item on its corresponding latent variable are between 0.65–0.882. As shown in Table 3, the average variance extracted (AVE) of each latent variable is greater than 0.5, and the combined reliability (CR) is greater than 0.8. The above indicators show that the scale of this study has good convergent validity.

#### 3.4.4. Discriminant Validity

As shown in Table 3, the square root of AVE value of each latent variable is greater than the correlation coefficient between this latent variable and other latent variables, indicating that each construct has both certain correlation and their own independence, so the discriminant validity of the scale in this study is good.

### 3.5. Common Method Deviation Test

Since the scale is used to measure the construct in this study, there may be a problem of common method deviation. We adopted a series of control procedures to reduce the interference caused by common method deviation, such as emphasizing the anonymity of this study, improving the items and order of the scale, etc. Referring to previous studies, this paper uses two methods to test whether there is a common method deviation. Firstly, Harman single factor test was carried out by using SPSS 25 0 to make exploratory factor analysis on each item. The results showed that the variance interpretation rate of the first non-rotating factor was 33.554%, less than 50%, so there was no significant common method deviation. Secondly, confirmatory factor analysis with common method factors was conducted [52]. The confirmatory factor analysis model M1 and the confirmatory factor analysis model M2 with common factors are constructed respectively, and the main fitness indexes of the two models are compared: $\Delta \frac{\chi^{2}}{df}=$ 0.039, ΔRMSEA = 0.003, ΔTLI = 0.003, ΔCFI = 0.003, all less than 0.05, indicating that the model fitness degree has not been significantly improved after adding the common method factor, so there is no significant common method deviation problem [53].

## 4. Data Analysis and Empirical Results

SPSS 25.0 and AMOS 24.0 are used to analyze the data in this study. First, the structural equation model includes both the path test model and the construct measurement model, which can estimate the parameters in the model as a whole. This method is very suitable for testing the relationship between multiple variables. In addition, the maximum likelihood method is a commonly used parameter estimation method. Therefore, the structural equation model is used in this study to test the relationship between variables, and the parameter estimation method is the maximum likelihood method. Secondly, bootstrapping uses the method of self-sampling to fit the probability distribution, which is suitable for hypothesis testing with unknown probability distribution. Therefore, we use the bootstrapping method to test the significance of the mediation effect value. Among them, the mediating effect test is conducted using the percentile Bootstrap method with deviation correction. The 95% confidence interval of the mediating effect value does not contain 0, indicating that the mediating effect is significant; otherwise, it is not significant. Finally, for the moderation effect test, the interaction term needs to be added to the regression model, so we use SPSS 25.0 to test the moderation effect.

### 4.1. Model Fitness and Path Coefficient Test

In order to explore the relationship and mechanism among community green interaction, consumer environmental emotion and consumer related green purchase behavior, AMOS 24.0 is used to test the data. The path test results and model fitness indicators are shown in Table 4. It can be seen from the Table 4 that the absolute fitness index $\frac{\chi^{2}}{df}$ = 1.248 < 3, RMSEA = 0.027 < 0.05, GFI = 0.921, AGFI = 0.906, which are all greater than 0.9. Value added fitness index NFI = 0.926, RFI = 0.918, TLI = 0.983, CFI = 0.984, which are all greater than 0.9. Therefore, the fitness degree of the model is good.

It can be seen that green information interaction has a significant positive impact on related green purchase behavior (β = 0.139, *p* = 0.009 < 0.05, C.R. = 2.631 > 1.96); green interpersonal interaction has a significant positive impact on related green purchase behavior (β =0.119, *p* = 0.019 < 0.05, C.R. = 2.336 > 1.96), indicating that Hypothesis H1a and H1b passed the test. Green information interaction has a significant positive impact on positive environmental emotion (β = 0.332, *p* = 0.000 < 0.05, C.R. = 6.561 > 1.96), and also has a significant positive impact on negative environmental emotion (β = 0.349, *p* = 0.000 < 0.05, C.R. = 6.253 > 1.96), indicating that H2a and H2b passed the test. Green interpersonal interaction has a significant positive impact on positive environmental emotion (β = 0.292, *p* = 0.000 < 0.05, C.R. = 5.945 > 1.96), and also has a significant positive impact on negative environmental emotion (β = 0.306, *p* = 0.000 < 0.05, C.R. = 5.608 > 1.96), showing that H3a and H3b are confirmed. Positive environmental emotion has a significant positive impact on related green purchase behavior (*β* = 0.32, *p* = 0.000 < 0.05, C.R. = 4.466 > 1.96). Similarly, negative environmental emotion has a significant positive impact on related green purchase behavior (*β* = 0.278, *p* = 0.000 < 0.05, C.R. = 4.557 > 1.96), indicating that H4a, H4b, H4c, H4d are partially supported.

### 4.2. Test of Mediating Effect of Environmental Emotion

In order to further test the mediating effect of positive environmental emotion and negative environmental emotion, this study adopted the Bootstrap mediating effect test method proposed by Preacher and Hayes [54] and adopted the Bias-corrected percentile Bootstrap method according to Fang et al. [55], using AMOS 24.0 for repeated sampling 5000 times to calculate the 95% confidence interval of the mediating effect.

As Table 5 shows, positive environmental emotion and negative environmental emotion play a mediating role in the impact of community green information interaction on related green purchase behavior. Among them, the mediation effect value of positive environmental emotion is 0.106, with the confidence interval being [0.057,0.175], excluding 0, indicating that the mediating effect is significant, and H4a is confirmed. The mediating effect value of negative environmental emotion is 0.097, with the confidence interval being [0.054,0.159], excluding 0, indicating that the mediating effect is significant, and H4b is confirmed. It can be seen from Table 4 that the direct effect of community green information interaction on related green purchase behavior is significant, accounting for 40.64%, while the mediating effect of positive environmental emotion and negative environmental emotion accounts for 30.99% and 28.36%, respectively. Therefore, both positive environmental emotion and negative environmental emotion play a partial mediating role in the path of community green information interaction-related green purchase behavior.

In the impact of community green interpersonal interaction on related green purchase behavior, positive environmental emotion, and negative environmental emotion also play a mediating role. Among them, the mediating effect value of positive environmental emotion is 0.094, with the confidence interval being [0.047,0.16], excluding 0, indicating that the mediating effect is significant, and H4c is confirmed. The mediating effect value of negative environmental emotion is 0.085, with the confidence interval being [0.043,0.144], excluding 0, indicating that the mediating effect is significant, and H4d is confirmed. Similarly, the direct effect of community green interpersonal interaction on related green purchase behavior is significant, accounting for 39.93%, while the mediating effect of positive environmental emotion accounted for 31.54%, and the mediating effect of negative environmental emotion accounted for 28.52%. Therefore, both positive environmental emotion and negative environmental emotion play a partial mediating role in the path of community green interpersonal interaction-related green purchase behavior.

### 4.3. Test of Moderating Effect of Product Involvement

This study uses SPSS 25.0 to verify the moderating effect of product involvement by using multi-layer regression analysis method, and constructs Models 1 to 6. The results are shown in Table 6.

**Figure 2:**
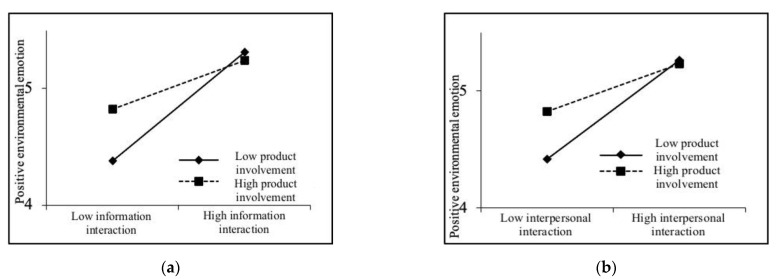
Moderating effect of product involvement. (**a**) Moderating effect of product involvement on the path “green information interaction—positive environmental emotion”. (**b**) Moderating effect of product involvement on the path “green interpersonal interaction—positive environmental emotion”.

Firstly, the moderating effect of product involvement on the path of “community green interaction—positive environmental emotion” was examined. The results of Model 3 showed that community green information interaction and community green interpersonal interaction have a significant positive impact on positive environmental emotion, and the explanatory power of the model is improved (R2 is improved) after the interaction item was added. The interaction coefficient of product involvement and community green information interaction is −0.130 (*p* < 0.05), and the interaction coefficient of product involvement and community green interpersonal interaction is −0.112 (*p* < 0.05), that is, product involvement has a significant negative moderating effect between community green interaction and positive environmental emotion (as shown in Figure 2), indicating that H5a and H5b are supported.

**Figure 3:**
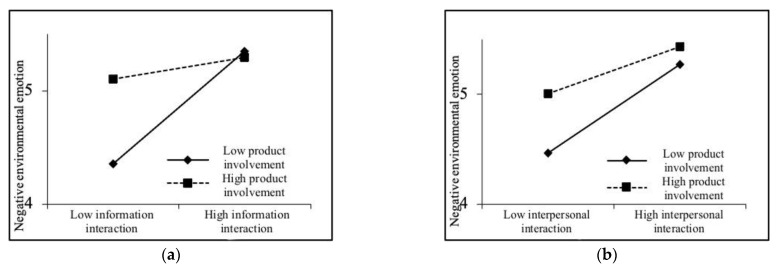
Moderating effect of product involvement. (**a**) Moderating effect of product involvement on the path “green information interaction—negative environmental emotion”. (**b**) Moderating effect of product involvement on the path “green interpersonal interaction—negative environmental emotion”.

Secondly, the moderating effect of product involvement on the path of “community green interaction—negative environmental emotion” was examined. As shown in Model 6, the explanatory power of the model is improved (R^2^ is improved) after the interaction item was added. The interaction item coefficient of product involvement and community green information interaction is −0.200 (*p* < 0.05), and the interaction item coefficient of product involvement and community green interpersonal interaction is −0.097 (*p* < 0.05), which shows that product involvement has a significant negative moderating effect on the path of “community green interaction-negative environmental emotion” (as shown in Figure 3). Thus, H5c and H5d are verified.

This study further used the Johnson-Neyman method to make Floodlight Analysis on the moderating effect. The results showed that in the path of “community green information interaction-positive environmental emotion”, community green information interaction has a significant impact on positive environmental emotion when the level of product involvement is lower than 6.729. In the path of “community green information interaction- negative environmental emotion”, community green information interaction has a significant impact on negative environmental emotion when the level of product involvement is lower than 6.189. In the path of “community interpersonal information interaction-positive environmental emotion”, community green information interaction has a significant impact on positive environmental emotion when the level of product involvement is lower than 6.912. In the path of “community green information interaction- negative environmental emotion”, green information interaction has a significant impact on negative environmental emotion when the level of product involvement is lower than 6.546.

## 5. Discussion

Based on cognitive dissonance theory, self-perception theory, and social diffusion theory, combined with the research paradigm of “interaction-psychological change-response”, this study puts forward the hypothesis model of “interaction-emotion-behavior” spillover effect and empirically tests the applicability, explanatory power, and boundary conditions of the spillover effect model of community green interaction on related green purchase behavior. When consumers interact on the brand community or network platform, this kind of community green interaction will drive more people to participate in green consumption, that is, the influence of community green interaction in promoting the diffusion of green consumption in the whole society is becoming increasingly prominent with the dissemination and diffusion of green consumption information. This study analyzes the phenomenon that community green interaction spills over to related green purchase behavior and shows that consumers’ “former green” in one field can effectively drive the “latter green” in other fields and even the “common green” in the whole field. This study also reveals the generation mechanism and underlying causes of the spillover effect of community green interaction. The core idea of this study is that community green interaction (consumers generate “first green “ in a certain field, which is the breakthrough) can bring about the improvement of green cognition and community belonging, which will further affect consumers’ environmental emotion. After consumers have higher environmental emotion, they will be more inclined to carry out related green purchase behavior in their daily life (that is, generate “latter green” in other fields and even “common green” in the whole field). Community green interaction helps to improve consumers’ environmental emotion and finally spills over to the follow-up or other green purchase behaviors in daily life, which is the social diffusion mechanism of community green interaction. Our results reveal several interesting phenomena as follows.

(1) Our research proves that community green interaction (including two dimensions of community green information interaction and community green interpersonal interaction) has a positive spillover effect on consumers’ related green purchase behavior. This is in line with previous studies which argued that social interaction indeed influences consumer buying behavior. Adjei et al. (2010) pointed out that online C2C communication has positive influence on immediate purchase intentions, and the depth and breadth of future purchase [34]. Jing and Yu (2020) found that online brand community interaction significantly improves consumers’ purchase intention [35]. However, the purchase behavior referred to in past research is a broad concept.

Our research further refines consumers’ purchasing behavior, focusing on consumers’ related green purchasing behavior. Further, we divide community green interaction into two dimensions: community green information interaction and community green interpersonal interaction. Our study demonstrates that both have positive spillover effects on consumer-related green buying behavior. We believe that the reason for this spillover effect is the improvement of cognition and identity based on community interaction. Community green information interaction can effectively enhance consumers’ green awareness. While community green interpersonal interaction can effectively enhance consumers’ sense of identity and belonging.

Li (2011) believes that group identity will be generated after experiencing social activities with common cultural atmosphere with members, interaction between members and website, and then further contact activities [56]. To some extent, this also reflects the idea of Bandura’s Social learning theory, that is, human behavior is the product of the interaction of internal processes and external influences. Khare et al. (2021) found that online communities and celebrities significantly predicted green clothing purchase behavior [57].

Liu and Liu (2020) showed that information interaction and interpersonal interaction in virtual brand community have significant positive effects on impulse buying [58]. It shows that community interaction does have an impact on consumer purchasing behavior. We believe that if the theme of consumer interaction is related to environmental protection, then it will help promote their green buying behavior. Therefore, how to effectively guide consumers to engage in green community interaction is a key issue.

(2) Secondly, this study proves that community green interaction (two dimensions of green information interaction and green interpersonal interaction) has a positive effect on consumers’ environmental emotion (including positive environmental emotion and negative environmental emotion). This is because green information interaction can improve consumers’ green cognitive level, and the improvement of cognition can enhance their emotion. Previous research has proved that increased awareness increases consumer’s environmental emotion [39]. Ye (2019) showed that the improvement of green awareness can lead to the improvement of consumers’ green emotion [57]. The reason why community interpersonal interaction can improve environmental emotion is that consumers have identity [45] and emotional belonging to their community after participating in interpersonal interaction, which is more vulnerable to the influence of other members’ concept of green environmental protection. Trust, identification and interaction factors in online communities can trigger consumers’ cognitive and emotional responses. Frequent communication and interaction among members can mobilize consumers’ positive emotions and product cognition [56]. The behavior of others can also become the reference of their own behavior, and the environmental emotion has been enhanced imperceptibly. This shows that the antecedent of environmental emotion is not only cognition, but also behavior, which is an important supplement to the previous studies.

Secondly, both positive and negative environmental emotion positively affect consumers’ related green purchase behavior, and positive environmental emotion has a stronger impact on related green purchase behavior (β_Path coefficient of positive environmental emotio__n_ = 0.32 > β_Path coefficient of negative environmental emotion_ = 0.278), which is consistent with the conclusions of previous studies [40,59]. Our study proves once again the applicability and extensibility of the broaden-and-build theory of positive emotions proposed by Fredrickson [60] in the field of green consumption, that is, compared with negative emotions, positive emotions are more helpful for individuals to make appropriate behavior choices. This study argues that the reason may be that consumers will avoid negative environmental emotion in order to maintain a positive self. Therefore, compared with positive environmental emotion, negative environmental emotion has less effect. Some recent studies, such as Khan and Mohsin (2017) and Joshi et al. (2021), showed that positive emotions act as a powerful driving factor for green purchase behavior [61,62].

In summary, Community green interaction can positively spillover to consumers’ related green purchase behavior through the psychological path of environmental emotion. In the two paths of community, green information interaction-related green purchase behavior and community green interpersonal interaction-related green purchase behavior, both positive environmental emotion and negative environmental emotion are partial mediating variables, and there is no significant difference in the mediating effect value. Positive environmental affect and negative environmental affect can play a mediating role at the same time, which is consistent with previous research conclusions [46,47,48]. It is very important that environmental emotion is different from emotion, which is a stable psychological variable. Therefore, our conclusions help shape consumers’ green buying behavior and green buying habits. Combined with the above discussion, the theoretical model of this study can be summarized as “interaction-emotion-behavior”, that is, interaction can improve environmental emotion and further spillover to subsequent behavior. On the one hand, it reflects the dynamic relationship and influence mechanism between behavior and behavior; on the other hand, it is also the development of previous theoretical models.

(3) Product involvement has a significant negative moderating effect on the path of “community green interaction—environmental emotion”. That is to say, both community green information interaction and community green interpersonal interaction have a great effect on the environmental emotion of consumers with low product involvement, while the effect on the environmental emotion of consumers with high product involvement is weak. This shows that “community green interaction—environmental emotion” is not applicable to all consumers. Consumers with high product involvement tend to use the central path to process information [63], so they pay more attention to the professional information and knowledge they can get in the community interaction. Therefore, community green interaction has a weak effect on their environmental emotion. However, consumers with low product involvement use edge path to process information [64,65], they are more likely to have emotional changes after participating in community interaction. This is consistent with Areni (2003) and Liu et al. (2015), that consumers with low product involvement pay more attention to emotional and transformational information, while consumers with high product involvement pay more attention to rational and functional information [53,66].

## 6. Conclusions

This study focuses on the direction, mechanism and boundary conditions of the spillover effect of community green interaction on consumer’s related green purchase behavior. The main conclusions of this paper are as follows:

(1) Community green information interaction and community green interpersonal interaction have a significant impact on consumers’ related green purchase behavior;
(2) Community green interaction (two dimensions of green information interaction and green interpersonal interaction) has a positive effect on consumers’ environmental emotion (two dimensions of positive environmental emotion and negative environmental emotion);
(3) Both positive and negative environmental emotion positively affect consumers’ related green purchase behavior;
(4) Community green interaction can positively spillover to consumers’ related green purchase behavior through the psychological path of environmental emotion;
(5) Product involvement has a significant negative moderating effect on the path of “community green interaction—environmental emotion”.

### 6.1. Theoretical Contributions

The main theoretical contributions of this study are as follows:

First, it fills the lack of research on the application of community interaction in the field of green consumption, enriches the research on spillover effect, and finds the social diffusion mechanism of community green interaction. Existing studies generally examined the role of community interaction in products, industries, after-sales services, and other fields, and few studies pay attention to community green interaction and its impact on follow-up and other related behaviors. For the first time, this study selects “little bear fuel consumption community” as the research object, and empirically tests the spillover effect and social diffusion mechanism of community green interaction on related green purchase behavior in the Chinese context.

Second, this study proposes that community green interaction is divided into two dimensions: community green information interaction and community green interpersonal interaction, which provides more space and possibilities for follow-up research. From the perspective of outcome variables, past studies often regarded psychological variables such as consumer loyalty, satisfaction and purchase intention as the outcome variables of community interaction. This study explored the dynamic relationship between the two coherent behaviors of community green interaction and green purchase, which is a supplement to the research paradigm of community interaction.

Third, this study constructs and tests the theoretical model of “community green interaction—environmental emotion—related green purchase behavior”, and further confirms that community green interaction can spillover to related green purchase behavior through the path of environmental emotion from the perspective of environmental emotion, which opens the “black box” of the diffusion mechanism of community green interaction and provides a new perspective for the explanation of spillover effect. In addition, this study reveals the negative moderating effect of consumer product involvement on the path of “community green interaction environmental emotion”, and clarifies the boundary conditions of the theoretical model of this study. This study enriches the research on the antecedents of product involvement and consumer environmental emotion and expands the application of relevant theories.

### 6.2. Management Implications

First, it is proposed that community members should be guided to actively participate in community green information interaction. This study shows that professional green information interaction in the community can have a positive spillover effect on related green purchase behavior. Therefore, the government should encourage various enterprises and network platforms to actively build various forms of online or offline green communities, such as online WeChat groups, discussion groups, green communities, and offline green teams, to provide consumers with a platform for information interaction. In the community, professional green information can be published regularly, such as knowledge related to enterprise green products, popular science videos, usage tips, etc. On the one hand, it can stimulate members’ intentions to participate in green information interaction; on the other hand, this is also a way for enterprises to export environmental protection values, which can not only enhance the image of enterprises, but also publicize their green products, make the content of green information interaction more closely related to the purchase behavior of green products, and give full play to its positive spillover effect on the related green purchase behavior. In addition, various answer competitions on green, green product experience exchange and green knowledge sharing meetings can be regularly organized within the community, and certain rewards can be given to enhance the enthusiasm of community members to participate in green information interaction.

Second, guide community members should be guided to actively participate in community green interpersonal interaction. This study shows that green interpersonal interaction in the community can have a positive spillover effect on related green purchase behaviors, such as discussing oil prices, exchanging daily environmental behaviors, etc. Therefore, enterprises should strengthen the guidance of community green interpersonal interaction. Governments and enterprises can regularly push some current hot environmental topics, such as “is plastic a great invention or a bad invention?” “Do you choose to order takeout without tableware?” and guide members to discuss within the community, so as to strengthen the interpersonal interaction among community members. In addition, enterprises should also maintain a good community interaction atmosphere, and can select members with strong environmental awareness as the main managers of the community. On the one hand, they have the ability to call on other members of the group to join the interaction and can play a “catalyst” role in the process of green interpersonal interaction in the community. On the other hand, group management by community members can create a more relaxed and pleasant interactive atmosphere, help to improve members’ sense of participation and emotional belonging, and give better play to the spillover effect of community green interpersonal interaction on related green purchase behavior.

Third, the connection of environmental emotion should be established through community interaction. Environmental emotion is the intermediate mechanism of the spillover effect of community green interaction on related green purchase behavior, which should be paid attention to by enterprises. On the one hand, enterprises can convey the benefits of green environmental protection to individuals and society through video, pictures, text, and other forms; trigger discussions among community members; and then improve the positive environmental emotion of community members. On the other hand, enterprises can appropriately show community members the bad environmental behaviors existing in the current society and the harm caused by these behaviors to the environment and individuals, and trigger members’ discussion, so as to improve the negative environmental emotion of community members. Through the above methods, consumers’ emotional experience in the process of community green interaction should be strengthened, and consumers’ subsequent green purchase behavior should be further stimulated. In addition, in view of the stronger impact of positive environmental emotion, enterprises can appropriately increase the transmission of positive emotion.

Fourth, consumers with different product involvement should be distinguished and the spillover effect of community green interaction should also be given better play to. Community green interaction has different effects on consumers with high product involvement and low product involvement. Therefore, enterprises should distinguish consumers with different product involvement and guide them accordingly. Specifically, enterprises can divide consumers with different product involvement into several groups. For consumers with low product involvement, enterprises can regularly push emotion oriented green information and environmental protection videos, so as to better play the role of community interaction in improving environmental emotion, and impose the spillover effect on related green purchase behavior. For consumers with high product involvement, community green interaction has little impact on environmental emotion, so professional green information related to green products, industries and energy conservation, and emission reduction can be pushed to stimulate their interest in participating in community interaction and strengthen the direct spillover of community green interaction on related green purchase behavior.

### 6.3. Research Limitations and Future Prospects

There are still some limitations in this paper, which is worthy of further research in the future. This study concludes that consumers’ positive environmental emotion and negative environmental emotion have partial mediating effects, and some mechanisms have not been explored, which is worthy of further exploration. Is the community green interaction environmental emotion related green purchase behavior model proposed in this paper applicable to a wider range of samples? Are there other boundary conditions? Can the “interaction-emotion-behavior” model proposed in this paper be applied to explain a wider range of phenomena? Can it be integrated with the traditional “knowledge-emotion-action” model? The above problems need to be further solved and improved in the future.

## Figures and Tables

**Table 1:** Measurement items of the scale and results of confirmatory factor analysis.

Latent Variable	Item	Standardized Factor
1 Community green information interaction	I will discuss fuel consumption records in the community	0.722
	I will discuss how to save fuel consumption in the community	0.807
	I will exchange information about cars with low consumption in the community	0.776
	I will exchange information about new energy vehicles in the community	0.878
	I will exchange the trend of the automobile industry in the community	0.756
2 Community green interpersonal interaction	I will discuss environmental behavior in the community	0.669
	I will discuss oil price information in the community	0.731
	I will discuss daily traffic conditions in the community	0.705
	I think members of the community and I trust each other	0.859
	I think I have established a friendship with members of the community	0.789
3 Positive environmental emotion	I feel happy to contribute to environmental protection by getting information on fuel conservation	0.735
	I feel happy to get information about environmental protection and contribute to environmental protection.	0.656
	I feel happy that I can contribute to environmental protection by practicing environmental behavior every day.	0.693
	I commend my members for saving fuel consumption and contributing to environmental protection	0.797
	I commend members for their contribution to environmental protection by using environmentally friendly car accessories	0.664
	I applaud the members for their daily practice of environmental protection	0.762
4 Negative environmental emotion	I feel guilty for not saving fuel consumption and causing harm to the environment	0.735
	I feel guilty for using resource consuming auto parts to destroy the environment	0.854
	I feel guilty for the harm caused by my daily environmental damage	0.780
	I am worried that non community members do not save fuel consumption and do harm to the environment	0.805
	I’m worried that non community members use consumable accessories to damage the environment	0.728
	I am worried about the harm caused by environmental damage by non-members of the community	0.714
5 Related green purchase behavior	I’m willing to buy a car with less fuel consumption	0.711
	I am willing to buy new energy vehicles	0.835
	I am willing to buy environmental protection products in my daily life	0.879
	I would like to recommend my friends to buy cars with less fuel consumption consumption	0.687
	I would like to recommend my relatives and friends to buy new energy vehicles	0.653
	I would like to recommend relatives and friends to buy environmental protection products in daily life	0.806
6 Product involvement	I will spend time learning about the cars I buy	0.880
	Information about cars in community interaction is what I need	0.823
	The information about cars in community interaction is valuable to me	0.736
	The information about saving fuel consumption in community interaction is what I need	0.805

**Table 2:** Descriptive statistics of samples.

Demographics	Category	Number	Percentage
Gender	Male	222	63.8%
	Female	126	36.2%
Age	19–24 years old	89	25.6%
	25–34 years old	144	41.4%
	35–44 years old	72	20.7%
	45–55 years old	24	6.9%
	Over 55 years old	19	5.5%
Education	Junior school or below	19	5.5%
	Senior school or technical secondary school	28	8.0%
	College or vocational school	105	30.2%
	Undergraduate	152	43.7%
	Postgraduate and above	44	12.6%
Monthly income	Below 3500 yuan	74	21.3%
	3501–5000 yuan	67	19.3%
	5001–6500 yuan	81	23.3%
	6501–8000 yuan	47	13.5%

**Table 3:** Analysis results of correlation coefficient, reliability and discriminant validity.

Latent Variable	1	2	3	4	5	6
1 Community green information interaction	0.789					
2 Community green interpersonal interaction	0.319	0.754				
3 Positive environmental emotion	0.501	0.471	0.74			
4 Negative environmental emotion	0.471	0.442	0.546	0.77		
5 Related green purchase behavior	0.484	0.453	0.593	0.578	0.766	
6 Product involvement	0.283	0.147	0.295	0.334	0.353	0.812
Cronbach’salpha	0.89	0.866	0.875	0.896	0.892	0.885
AVE	0.623	0.568	0.547	0.594	0.587	0.660
CR	0.892	0.867	0.878	0.897	0.894	0.886

Note: the diagonal value is the square root of AVE, and the lower left part is the Pearson correlation coefficient between latent variables.

**Table 4:** Path test results and model fitness indicators.

Path			Non-Standardized Coefficient	Standardized Coefficient	S.E.	C.R.	P
Community green information interaction→ Positive environmental emotion			0.332	0.398	0.051	6.561	***
Community green information interaction→ Negative environmental emotion			0.349	0.377	0.056	6.253	***
Community green interpersonal interaction→ Positive environmental emotion			0.292	0.356	0.049	5.945	***
Community green interpersonal interaction→ Negative environmental emotion			0.306	0.335	0.054	5.608	***
Community green information interaction→ Related green purchase behavior			0.139	0.157	0.053	2.631	0.009
Community green interpersonal interaction→ Related green purchase behavior			0.119	0.136	0.051	2.336	0.019
Positive environmental emotion→ Related green purchase behavior			0.32	0.302	0.072	4.466	***
Negative environmental emotion→ Related green purchase behavior			0.278	0.292	0.061	4.557	***
$\chi^{2}/df$	RMSEA	GFI	AGFI	NFI	IFI	TLI	CFI
1.248	0.027	0.921	0.906	0.926	0.984	0.983	0.984

Note: *** *p* < 0.001.

**Table 5:** Mediating effect test by Bootstrap method.

Path	Mediator	Effect Value	SE	Bias-Corrected 95%CI		
				Lower	Upper	*p*
Community green information interaction---- related green purchase behavior	Positive environmental emotion	0.106	0.029	0.057	0.175	***
	Negative environmental emotion	0.097	0.026	0.054	0.159	***
Community green interpersonal interaction ----related green purchase behavior	Positive environmental emotion	0.094	0.028	0.047	0.16	***
	Negative environmental emotion	0.085	0.025	0.043	0.144	***

Note: *** *p* < 0.001.

**Table 6:** Moderating effect of product involvement.

Variable	Positive Environmental Emotion			Negative Environmental Emotion		
	Model 1	Model 2	Model 3	Model 4	Model 5	Model 6
(Constant)						
Gender	0.131 **	0.131 **	0.115 **	0.192 ***	0.190 ***	0.171 ***
Age	−0.047	−0.044	−0.064	0.056	0.062	0.036
Education	0.182 ***	0.173 ***	0.184 ***	0.183 ***	0.166 ***	0.174 ***
Community green Information interaction	0.368 ***	0.346 ***	0.337 ***	0.351 ***	0.311 ***	0.295 ***
Community green interpersonal interaction	0.327 ***	0.320 ***	0.312 ***	0.327 ***	0.315 ***	0.309 ***
Product involvement		0.096 ***	0.096 *		0.175 ***	0.176 ***
Product involvement × Community green information interaction			−0.130 **			−0.200 ***
Product involvement × Community green interpersonal interaction			−0.112 *			−0.097 *
R^2^	0.363	0.372	0.406	0.348	0.376	0.433
Adjusted R^2^	0.354	0.361	0.392	0.339	0.365	0.419
F value	39.011	33.624	28.997	36.534	34.307	32.321

Note: * *p* < 0.05; ** *p* < 0.01; *** *p* < 0.001.

## Data Availability

Some or all data and models that support the findings of this study are available from the corresponding author upon reasonable request.
